# Echovirus type 6 transmission clusters and the role of environmental surveillance in early warning, the Netherlands, 2007 to 2016

**DOI:** 10.2807/1560-7917.ES.2018.23.45.1800288

**Published:** 2018-11-08

**Authors:** Susana Monge, Kimberley Benschop, Loes Soetens, Roan Pijnacker, Susan Hahné, Jacco Wallinga, Erwin Duizer

**Affiliations:** 1 Centre for Infectious Disease Control (CIb), National Institute for Public Health and the Environment (RIVM), Bilthoven, the Netherlands; 2 European Programme for Intervention Epidemiology Training (EPIET), European Centre for Disease Prevention and Control, (ECDC), Stockholm, Sweden; 3 Department of Biomedical Data Sciences, Leiden University Medical Center, Leiden, The Netherlands

**Keywords:** enterovirus, environmental surveillance, E6, echovirus 6, cluster detection

## Abstract

**Background:**

In the Netherlands, echovirus type 6 (E6) is identified through clinical and environmental enterovirus surveillance (CEVS and EEVS).

**Aim:**

We aimed to identify E6 transmission clusters and to assess the role of EEVS in surveillance and early warning of E6.

**Methods:**

We included all E6 strains from CEVS and EEVS from 2007 through 2016. CEVS samples were from patients with enterovirus illness. EEVS samples came from sewage water at pre-specified sampling points. E6 strains were defined by partial VP1 sequence, month and 4-digit postcode. Phylogenetic E6 clusters were detected using pairwise genetic distances. We identified transmission clusters using a combined pairwise distance in time, place and phylogeny dimensions.

**Results:**

E6 was identified in 157 of 3,506 CEVS clinical episodes and 92 of 1,067 EEVS samples. Increased E6 circulation was observed in 2009 and from 2014 onwards. Eight phylogenetic clusters were identified; five included both CEVS and EEVS strains. Among these, identification in EEVS did not consistently precede CEVS. One phylogenetic cluster was dominant until 2014, but genetic diversity increased thereafter. Of 14 identified transmission clusters, six included both EEVS and CEVS; in two of them, EEVS identification preceded CEVS identification. Transmission clusters were consistent with phylogenetic clusters, and with previous outbreak reports.

**Conclusion:**

Algorithms using combined time–place–phylogeny data allowed identification of clusters not detected by any of these variables alone. EEVS identified strains circulating in the population, but EEVS samples did not systematically precede clinical case surveillance, limiting EEVS usefulness for early warning in a context where E6 is endemic.

## Background

Enteroviruses (EVs) are viruses of the *Picornaviridae* family mainly transmitted via the faecal–oral route, but also through eye, nose, and mouth secretions. Humans are the only reservoir of the four species classified A to D, within which more than 100 types have been described [1]. For public health purposes, EVs are mainly classified into polioviruses and non-polio EVs (NPEVs). As the polio eradication approaches, NPEVs are receiving increased attention as sources of morbidity and mortality [2,3], being the most common cause of viral central nervous system (CNS) infections [4-8]. They can present as sporadic cases or as clusters or even cause large outbreaks involving severe cases with neurological or respiratory complications [1-3]. Echovirus type 6 (E6, EV-B species) detections have been increasing in recent years in the Netherlands, where it caused 13% of all NPEV infections in 2016 [9,10]. It is one of the most frequently detected types circulating in Europe [8,11-13] and other regions [14-17], and has been implicated in meningitis outbreaks [10,18-21].

The World Health Organization [22] suggests three reasons for EV surveillance unrelated to polio eradication: to detect and respond to outbreaks, to establish disease burden data for public health planning, and to perform virological investigation and research. Serotyping and characterisation of circulating NPEVs, including rapid identification and close monitoring of emerging strains, is of public health relevance [23]. In the Netherlands, poliovirus surveillance is performed through clinical EV surveillance (CEVS), mainly in stool samples [9,24], complemented with environmental EV surveillance (EEVS) in sewage water [24]. Exclusion of poliovirus is based on virus isolation and sequencing of EV-positive samples. Results of both surveillance systems also provide valuable information on circulating NPEVs.

In the past, EEVS demonstrated to be more sensitive than surveillance based on clinical cases alone [24,25]. NPEVs isolates from EEVS are also phylogenetically related to clinical isolates, suggesting its potential for characterising the extent of NPEVs circulation and diversity [13,14,24,26,27] or recognising outbreaks [15,27]. More importantly, as demonstrated for poliovirus [28], NPEV infections have been preceded by silent circulation of the same strain in the environment [14,26,29,30], suggesting a role of EEVS in early warning. Incorporating data on time and space in the phylogenetic analysis could help investigate the relation between environmental and clinical NPEVs strains, and assess if EEVS captures ongoing transmission chains and could act as early warning.

The public health importance of E6, together with its detectability in sewage water [14,15,24], make it a good candidate to evaluate the full potential of environmental EV surveillance. Our objective was to identify transmission clusters of E6 in the Netherlands using time, place and phylogeny data of clinical and environmental strains, with the final goal of assessing the added value of environmental surveillance in general and, specifically, for early warning of E6 outbreaks.

## Methods

### Design and setting

We included all E6-positive clinical cases and environmental samples identified from 2007 through 2016 within CEVS and EEVS. Data from CEVS was anonymised. Both surveillance systems have been previously described [9,24,31]. Briefly, for the CEVS, samples from patients with EV-related illness are analysed at clinical laboratories. Laboratories are strongly advised to send positive samples to the National Institute for Public Health and the Environment (RIVM) or to a TypeNed laboratory for typing and exclusion of poliovirus [31]. RIVM predominantly receives samples from the central part of the Netherlands. EEVS is performed in sewage water from secondary schools or residential areas in regions at higher risk of poliovirus introduction, in the area known as the bible belt, where the uptake of vaccination is low because of religious beliefs [32]. In addition, ad hoc locations can be included, for example, downstream from asylum seeker centres [24] or from the residence of known shedders [33]. Samples are collected approximately every 6 weeks, varying by season or following polio alerts [24,33,34]. Samples from CEVS typed at the RIVM reasonably overlap with the areas covered by the EEVS.

### Laboratory methods

Laboratory procedures for the surveillance systems have been previously described in detail [24]. Briefly, RNA was isolated from sewage water cultures with positive cytopathic effect and 5’ UTR RT-PCR-positive clinical samples by automated extraction using the LC Nucleic Acid isolation kit (MagnaPure96, Roche, Almere, the Netherlands). RNA was eluted in 50 µL elution buffer and amplified in the semi-nested RT-PCR described by Nix et al. [35]. The sensitivity of this RT-PCR, defined as the equivalent of the lowest dose of cultured infectious virus detected by this RT-PCR, was 0.126 50% tissue culture infective dose (TCID50) for EV-B (prototype virus coxsackievirus B3). The 350–400 nt fragments of the VP1 gene were purified using ExoSAP-IT and sequenced at Baseclear (Leiden, the Netherlands). The partial VP1 sequences were edited using BioNumerics version 7.1 and used as input in the EV genotyping tool (http://www.rivm.nl/mpf/enterovirus/typingtool/), which has an automated algorithm to assign the species and (sub)type of the sequences entered [36].

### Data analysis

Distribution of E6 strains across time, place and phylogeny was described. Date of sample collection was used or, if missing, date of reception at RIVM. Samples from the same patient within 10 days were considered as a single episode. E6 strains were described by source (clinical or environmental) and month. Dot maps represented geographical location of strains as a random point within the polygon of the four-digit postcode (PC4) or, if missing, the municipality. For environmental samples, we used the location of the sewage sampling point and for clinical samples, the location of patients’ residence or, if missing, of the clinical laboratory.

Phylogenetic analysis was based on partial VP1 gene. The Enterovirus-B echovirus type 6 D’Amori strain GenBank no. AY302558 was used for sequence alignment using MEGA 7.0.21. Sequences were omitted if they were shorter than 270 nt or if they overlapped only partially with the fragment from position 2,611 to 2,881. Phylogenetic trees were built using the maximum-likelihood method with 1,000 bootstrap replicates and a threshold of 70%. Genetic diversity was assessed by pairwise distribution [37]. We used a genetic threshold of 15% to define subtype-specific phylogenetic clusters using Cluster Picker 1.2.4 [38].

We analysed E6 transmission clusters by applying the algorithm developed by Ypma et al. [39]. Briefly, it uses pairwise distances in time, place and phylogeny to calculate the relative distance in each dimension between any two cases (i.e. number of cases between any two cases in an ordered distance sequence) and multiplies the three dimensions to obtain a combined distance measure. A hierarchical clustering tree is constructed by sequentially connecting pairs of cases with the smallest distance, resulting in a clustering tree in which length of the branches represents the combined distance. Significance of the clusters given their height and size is calculated by bootstrapping methods [39]. In this study we considered clusters with p < 0.05. In addition, we chose a cut-off value of the tree height at 15% below which clusters are identified. This cut-off is set ad hoc and is guided by a plausibility assessment of the identified clusters, including the analysis of intra-cluster correlation and inter-case distances (algorithm available on github.com/lsoetens/ClusterViz/). Finally, we described the distribution of the identified transmission clusters across time, place and phylogeny. Analyses were performed using R 3.4.0.

### Accession numbers

VP1 partial sequences were deposited in GenBank; the accession numbers are listed in the Supplement.

## Results

### Description of samples from the clinical and environmental enterovirus surveillance

Between 2007 and 2016, RIVM received 1,067 sewage water samples from 25 locations within EEVS, and 3,728 samples from 3,506 clinical episodes in 3,452 patients within CEVS (Table). E6 was detected in 92 (8.6%) samples from EEVS. There was one sample with two distinct E6 viruses, therefore a total of 93 different E6 isolates were analysed. E6 was detected in 157 (4.5%) clinical episodes, hereafter referred to as cases. Of all episodes, 1,756 (50%) were male, 1,332 (38%) female and 418 (12%) of unknown sex, with similar proportions of E6 positivity (chi-squared test: p = 0.335). Median age (based on 3,124 episodes) was 3.1 months overall (interquartile range (IQR): 1.0 months–2.5 years) and 4.3 months in E6 cases (IQR: 0.9 months–18.3 years; Mann–Whitney U test: p = 0.339).

**Table:** Number of samples analysed within the clinical and environmental enterovirus surveillance systems, the Netherlands, 2007–2016 (n=4,795)

		Number of samples analysed		Samples positive for E6	
		n	% (column)	n	% (row)
CEVS: sample	Faecal	2,847	76.4	109	3.8
	Respiratory	403	10.8	6	1.5
	Cerebrospinal fluid	362	9.7	44	12.2
	Other	96	2.6	1	1.0
	Unknown	20	0.5	0	0.0
	**Total**	**3,728**	**100**	**160**	**4.3** ^ a^
EEVS: sampling point	13 villages	451	42.3	38	8.4
	11 schools	428	40.0	43	10.1
	1 asylum seeker centre	188	17.4	11	5.9
	**Total**	**1,067**	**100**	**92**	**8.6** ^ b^

CEVS: clinical enterovirus surveillance system; EEVS: environmental enterovirus surveillance system; E6: echovirus type 6.

^a^ Multiple samples allowed per patient, therefore the total number of positive samples is higher that total number of positive cases (160 positive samples in 157 cases).

^b^ In one sample two distinct E6 types were identified, therefore total number of distinct E6 strains isolated in the environment is 93.

### Distribution of echovirus type 6 strains across time, place and phylogeny

**Figure 1:**
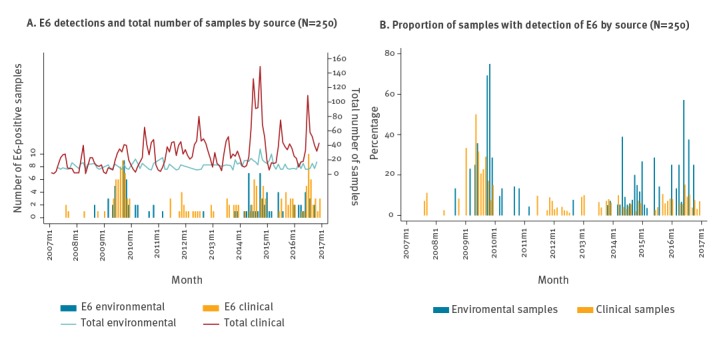
Echovirus type 6 detected in clinical (n = 157) and environmental surveillance (n = 93), the Netherlands, 2007–2016

E6 showed long periods of low incidence with peaks of increased circulation in 2009 and from 2014 onwards, compatible with an epidemic pattern (Figure 1). The number of EV-positive samples received for typing within the CEVS was variable, with peaks in the number of samples received coinciding with peaks in E6 detections. The variability in the number of sewage water samples was lower. To remove this interference, we calculated the proportion of samples positive for E6 in both systems. After doing that, the increase in 2014 was more evident in sewage than in clinical cases (Figure 1). No periodic pattern or long-term trend was evident. Importantly, there was no evidence that increases in environmental detections of E6 preceded the occurrence of clinical cases.

E6: echovirus type 6.

Panel A: Total number of E6 detections and total number of samples analysed per month from the environmental and the clinical surveillance systems. Panel B: Percentage of samples positive for E6 among the total number of samples by month.

**Figure 2:**
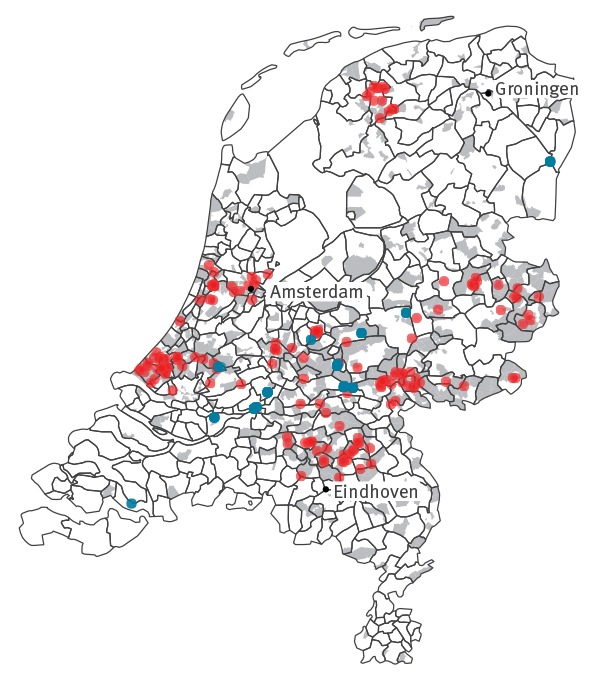
Geographical distribution of echovirus type 6 in clinical (n = 157) and environmental surveillance (n = 93), the Netherlands, 2007–2016

PC4 or municipality was available for all environmental surveillance locations and for 124 cases; for the remaining 33 cases, the PC4 of the virology laboratory was used. E6 cases were widely distributed in the central part of the country (Figure 2), matching the area covered by the CEVS and the EEVS. No evident geographical clustering was present overall, nor when looking at individual years (data not shown).

Red dots: clinical samples; blue dots: environmental samples. Boundaries represent municipalities, points represent random locations for cases within the 4-digit postcode polygon, and shaded grey areas represent 4-digit postcodes from which at least one sample was received in the clinical enterovirus surveillance system.

**Figure 3:**
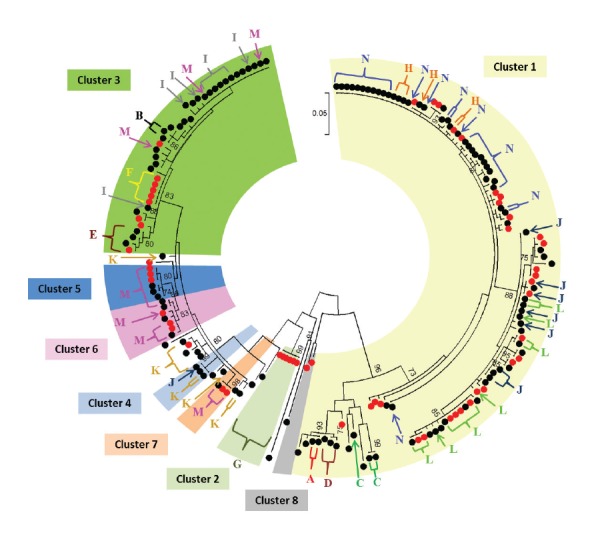
Phylogenetic tree of echovirus type 6 sequences in the VP1 positions 2,611–2,881, depicting eight identified phylogenetic clusters and 14 transmission clusters, the Netherlands, 2007–2016 (n = 188)

Partial VP1 sequences encompassing at least 270 nt were available for 188 isolates: 58 (62%) environmental and 130 (83%) clinical isolates. Eight phylogenetic clusters were identified (Figure 3 and Supplement Figure S1). Two contained only environmental isolates: cluster 2 (n = 6) and cluster 8 (n = 2) and one contained only clinical isolates: cluster 4 (n = 3). Five clusters included both: cluster 1 (31 environmental and 75 clinical isolates), cluster 3 (nine environmental and 30 clinical isolates), cluster 6 (three environmental and four clinical isolates), cluster 5 (three environmental and three clinical isolates) and cluster 7 (two environmental and one clinical isolate). There were 14 clinical isolates and two environmental isolates that did not belong to any phylogenetic cluster.

Maximum-likelihood method with 1000 bootstrap replicates, analysed in MEGA 7.0.21.

Red dots: echovirus type 6 isolates from environmental samples (n = 58) and black dots from clinical samples (n = 130). Background colours represent phylogenetic clusters. Letters represent transmission clusters.

**Figure 4:**
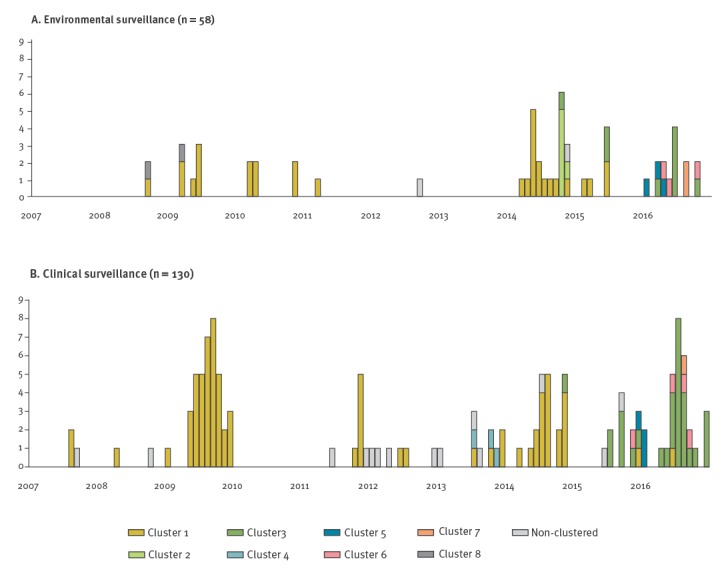
Monthly distribution of the eight phylogenetic clusters of echovirus type 6, the Netherlands, 2007–2016 (n = 188)

Cluster 1 was the dominant strain up to late 2014, with simultaneous detections in EEVS and CEVS (Figure 4). In 2015 and 2016, other phylogenetic clusters appeared. Cluster 3 appeared first in an environmental sample in October 2014, followed by a clinical case in November 2014, and again in the EEVS in June 2015 followed by the CEVS from July 2015 onwards. Cluster 5 appeared first in a clinical sample in December 2015 and was thereafter detected in other clinical cases and in the EEVS. Also cluster 6 was first detected in a clinical case in November 2015 and then sporadically during 2016 in other clinical cases and the EEVS. Cluster 7 appeared simultaneously in the CEVS and EEVS in August 2016. All phylogenetic clusters were geographically dispersed throughout the Netherlands (Supplement Figure S2).

### Transmission clusters

Using the time–place–phylogeny clustering algorithm, we identified 14 transmission clusters (designated A to N) comprising 122 (65%) isolates. Most transmission clusters were dense in time, place and genetics, with the exception of clusters J, K, N and M, which showed a significantly larger variation on the geographical dimension than the non-clustered isolates (however geographical distances in the Netherlands are small) (Supplement Figure S3). In addition, clusters C, D, E and H showed high intra-cluster correlation, indicating large internal cohesion in these clusters (Supplement Figure S4). For the other transmission clusters, the Spearman rank correlation coefficient between pairwise dimensions was either non-existent, very low, non-significant or could not be estimated because of zero variance in one or both dimensions.

**Figure 5:**
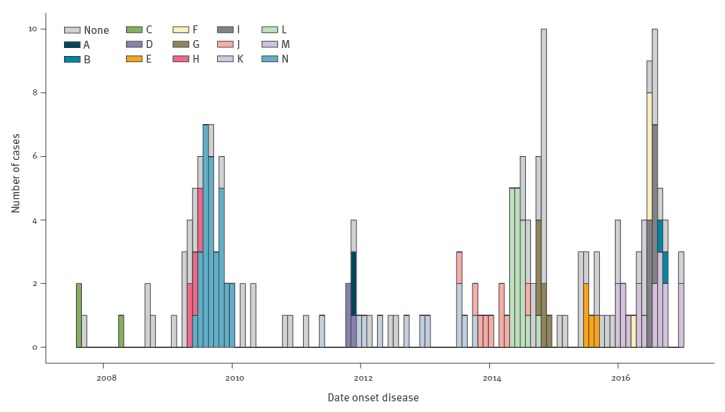
Monthly distribution of the 14 transmission clusters (A to N) of echovirus type 6, the Netherlands, 2007–2016 (n = 188)

Seven transmission clusters (A, C, D, H, J, L, N) were identified within phylogenetic cluster 1, and four (B, E, F, I) within phylogenetic cluster 3. Cluster N was the one responsible for the E6 peak of 2009, together with a few cases from transmission cluster H (Figure 5). Phylogenetic cluster 2 as a whole was identified as transmission cluster G and was the most robust cluster. Cluster K comprised isolates from phylogenetic cluster 4 along with isolates that were not part of a cluster based on phylogeny alone. Possible spurious clustering was identified by analysing within-cluster genetic distance (Supplement Table S1). In cluster J, very high genetic distance (21.5%) was identified between just one isolate (from phylogenetic cluster 4) and the rest of isolates (from phylogenetic cluster 1). After excluding this sequence, the maximum intra-cluster genetic distance was 1.3%. In cluster C, maximum distance was 8.3%, but decreased to 0% after the exclusion of one sequence. Cluster M, had pairwise distances higher than 1.5% (corresponding to 4 nt difference) in 78% of the pairs, corresponding to isolates from phylogenetic clusters 3, 5, 6 and 7. Cluster M also had the highest distances in phylogeny and time, spanning 12 months, as well as large geographical distance (Figure 5, Supplement Figures S4 and S6).

Six of the 14 transmission clusters included both environmental and clinical isolates (E, H, J–M), another six contained only clinical isolates (A–D, I, N) and two included only environmental isolates (F and G), although cluster G corresponded to the asylum seeker centre, for which no clinical information was available. Only in two (E and L) of the six transmission clusters including both clinical and environmental isolates the environmental E6 detection preceded the detection in CEVS.

## Discussion

In the Netherlands, E6 presented an epidemic pattern, with dispersed geographical distribution and increasing genetic diversity. Environmental and clinical samples belonged to joint phylogenetic clusters and, to a lesser extent, to joint transmission clusters, confirming the usefulness of environmental surveillance to characterise E6 strains causing disease in human populations. However, there was no indication that environmental detections systematically preceded detection through surveillance based on clinical cases.

To our knowledge, this is the first study to jointly analyse environmental and clinical samples of EVs using time, place and phylogeny to detect transmission clusters. Combining the three dimensions allowed identification of clusters not detected by any of these variables alone. Possible spurious clustering could be detected, probably explained by the simultaneous endemic circulation of different E6 strains in the same geographical region and time, as has been described by other authors [40]. It is not very likely that cluster M corresponded to a true transmission cluster, given the high genetic diversity of the isolates (more than 4 nt in 78% of the pairs), which fell within different phylogenetic groups, as well as the high inter-patient distances in time and in space. However, most of the transmission clusters fell within single phylogenetic clusters and were highly plausible. Separate transmission clusters of phylogenetically similar strains is most probably explained by two separate introductions of the same strain or by a circulating strain causing two distinct outbreaks. Our transmission clusters were also concordant with previous outbreak reports. Cluster N explained the period of increased E6 circulation in 2009. Interestingly, the nine clinical isolates identified as transmission cluster I corresponded to an outbreak that was detected between June and August 2016 and comprising 10 cases from the same province, of whom nine had neurological presentation; the 10th case from the outbreak was not in our study [10]. This supports the validity of our cluster detection method and highlights its usefulness for automated processing of complex data. Timely detection could potentially contribute to an earlier response to these clusters. Although there is no effective antiviral therapy against EVs, case management, enhanced hygiene measures and vigilance of close contacts can contribute to control and limit the spread of potentially severe EV infections.

At the frontier of polio elimination, also some other countries have implemented environmental EV surveillance [8,13,29,30]. Previous studies have demonstrated the potential of environmental specimens to contribute to the characterisation of circulating EVs in general and E6 in particular [14,41]. Environmental surveillance overcomes the reference bias present in clinical reports. While clinical surveillance only captures severe cases for whom physicians seek microbiological confirmation, environmental data provides a broader picture of circulating EVs. On the other hand, this could result in identification of strains with low virulence and low public health importance. The detection of novel EVs in the environment can indicate emerging strains that may cause outbreaks in the short or medium term. In Finland, close genetic relatives of the echovirus 30 strains that caused an epidemic in 2009 had already been isolated in the environment a few years before the outbreak [29]. A recent study in France found silent circulation of EV D68 in periods were no clinical cases were being reported [26] and in Greece, an E6 strain found in sewage water in 2006 was correlated with cases from an aseptic meningitis outbreak 1 year later [30]. This has raised the question of whether environmental surveillance could serve as early warning of increases in EV infections in the population.

Environmental surveillance was able to capture emerging strains causing disease in human populations, as 83% of all environmental isolates were clustered phylogenetically with clinical isolates. However, 45% did not belong to any transmission cluster and an additional 19% belonged to transmission clusters that did not include cases captured by clinical surveillance. Our results indicate that the usefulness of environmental surveillance for early warning of E6 is very limited, at least in contexts such as the Netherlands where E6 circulation is established and active typing of clinical samples is performed. The usefulness of environmental surveillance could potentially be improved by increasing sampling frequency, although the feasibility and cost-effectiveness of such a programme is questionable. Also, usefulness as early warning could be higher in countries where clinical surveillance is suboptimal or where E6 or other EV types are not endemic. For example, in Scotland, HPeV3 was not detected in the sewage sludge/sediment collected in Edinburgh in 2009 and only appeared in sewage one week before the first clinical case was diagnosed in early 2010 [13].

One of the transmission clusters detected the spread of E6 within an asylum seeker centre. The average stay in this centre was between 3 and 7 days, while in the EEVS, the cluster was detectable during 3 weeks, indicating subsequent transmission, rather than a unique shedder. Except in the sewage from the asylum seeker centre, this strain was not detected elsewhere, indicating that there was probably no transmission outside of this centre.

A limitation of our study is that the CEVS is based on voluntary referral of samples from clinical laboratories [31]. Moreover, there are no standard criteria for seeking microbiological confirmation of suspected EV cases, and this is done according to the expert opinion of the clinician in charge. This makes the system sensitive to variations in the frequency of EV testing, although we tried to control for this by analysing E6 as a proportion of all EV. Finally, since there are other laboratories in the Netherlands performing EV typing, not all EV clinical identifications in the Netherlands were captured by this study and the exact population coverage is not known. Environmental surveillance in the Netherlands is optimised for poliovirus exclusion in specific areas and may not provide a good representation of the general population, especially since the area covered (the bible belt) is considered to be relatively socially isolated and circulating strains can differ from other regions [24,32].

The VP1 fragment analysed in our study covered only 270 nt. While EV types can be assigned with this fragment, the fragment is too short to carry out a more in depth phylogenetic analysis. As such, the study only gives a first insight into possible clustering, which should be confirmed with complete VP1 sequencing. We used a 15% threshold for genetic homologues, which corresponds to the level of genetic variability observed in other studies. In Greece, in patients with CNS infection in 2006 and 2007, mean genetic distance between groups was 22% [8] and in Poland, including also environmental isolates, nucleotide sequence divergence was up to 19% [41]. As a limitation of the time–place–phylogeny clustering algorithm, we used 15% of the maximum distance as a threshold for cluster detection, and the number and composition of the resulting clusters is sensitive to this choice. Using 20% of the maximum distance or higher resulted in transmission clusters that were less consistent with the phylogenetic clusters previously defined, showed less plausibility and failed to identify the E6 cluster detected in 2016 [10].

## Conclusion

Transmission clusters are reliably identified by jointly analysing time, place and genetic information. Therefore, increasing efforts should be made to collect accurate time and place information within EV surveillance systems, along with genetic information. Automated algorithms can provide detection of such clusters and can potentially help control efforts to limit the spread of highly pathogenic EVs. Environmental surveillance proved to be valuable to characterise EVs, in this case E6, causing disease in human populations, but showed limited benefit for early warning for this endemic EV type.
